# Epigenetic and Genetic Factors Predict Women's Salivary Cortisol following a Threat to the Social Self

**DOI:** 10.1371/journal.pone.0048597

**Published:** 2012-11-14

**Authors:** Shany Edelman, Idan Shalev, Florina Uzefovsky, Salomon Israel, Ariel Knafo, Ilana Kremer, David Mankuta, Marsha Kaitz, Richard P. Ebstein

**Affiliations:** 1 Department of Neurobiology, Hadassah-Hebrew University Medical Center, Jerusalem, Israel; 2 Department of Psychology, Hebrew University, Jerusalem, Israel; 3 Technion Medical School, Haifa and Mazra Hospital, Akko, Israel; 4 Department of Labor and Delivery, Hadassah Medical Organization, Jerusalem, Israel; 5 Department of Psychology, National University of Singapore, Singapore, Singapore; Morehouse School of Medicine, United States of America

## Abstract

Evidence suggests that the reactivity of the Hypothalamus-Pituitary-Adrenal axis (HPAA) is modulated by both genetic and environmental variables. Of special interest are the underlying molecular mechanisms driving gender differences to psychosocial stressors. Epigenetic mechanisms that sculpt the genome are ideal candidates for mediating the effects of signals on the HPAA. In the current study, we analyzed by pyrosequencing, bisulfite-treated buccal DNA from male and female university students who participated in the Trier Social Stress Test (TSST). A linear regression model was used to ascertain the effects of sex, CpG methylation and genes on stress response. Total cortisol output (area under the curve, AUC) was significantly predicted by glucocorticoid receptor (*NR3C1*) exon 1F methylation (averaged across 39 CpG sites) solely in female subjects. A single CpG site located in the exon 1F noncanonical nerve growth factor-inducible protein A (NGFI-A) transcription factor was a highly significant predictor of AUC in female subjects. Additionally, variations in the estrogen receptor alpha (ESR1) and the serotonin transporter promoter (5-HTTLPR) genes were independent additive predictors of AUC. The full model accounted for half of the variance (50.06%) in total cortisol output. Notably, this is the first demonstration that epigenetic changes at the GR exon 1F correlate with HPAA reactivity. These findings have important implications for understanding the molecular mechanisms underlying gender differences in stress-related disorders and underscore the unique value of modeling both epigenetic and genetic information in conferring vulnerability to stress.

## Introduction

Psychosocial stress along with the coping styles that people employ when challenged by stress, are considered important determinants of overall wellbeing [1]. Particularly important is the body's reaction to social stressors; reflecting the daily changes we face at home, with friends, during school and at work. Dickerson & Kemeny [2] review evidence that human cortisol responses to acute stressors are most pronounced in situations that pose a social threat to the individual. Notably, not all individuals respond similarly to social stress and as noted by McEwen [3] there are very large individual differences in stress reactivity, reflecting significant life events. While some individuals appear to be resilient to difficult conditions, others react adversely to such challenges, incurring a range of physical and mental disorders. This imbalance is expressed via dysregulation of the Hypothalamus-Pituitary-Adrenal axis (HPAA), the main pathway of the stress response (with cortisol acting as its end product). The HPAA has been meticulously investigated and found connected to a large number of stress related disorders [4].

One of the most consistent findings employing psychological stress tasks is the significantly larger salivary cortisol response in healthy adult men compared to women following short-term laboratory stress [5], [6], [7], [8], [9]. Kudielka and Kirschbaum [10] extensively reviewed gender differences in response to stress and suggest that such differences reflect causal links and are not merely epiphenomena of sex-specific prevalence rates of several disorders [11], [12]. Male stress responses may predominantly involve the traditional “fight and flight” reaction while women's stress response may be better characterized by “tend and befriend”, involving nurturant activities and the creation of social networks. Differences between genders in stress response can be attributed to circulating gonadal sex hormones, sexual dimporphism of brain functioning and corticosteroid binding [13]. However, much of the underlying neurochemical and neurogenetic mechanisms for gender differences in stress reactivity generally remain obscure.

The final target of the HPAA cortisol release is the glucocorticoid receptor (GR, *NR3C1* MIM +138040) [14]. The GR is a member of the steroid receptor superfamily and is the key mediator of the majority of cortisol's tissue effects by way of direct binding to hormone-responsive elements in the DNA or via interactions with other transcription factors and regulation of gene transcription [14]. GR levels are transcriptionally controlled by multiple untranslated alternative first exons, each with its own promoter providing a mechanism for tissue-specific fine-tuning of GR levels [15]. The mechanisms regulating the differential usage of these first exons in different tissues and individuals, and the role of the 5′-UTR in the splicing of the coding exons are still poorly understood (see review by Turner et al [16]). Both individual and gender differences are observed in basal and dynamic cortisol changes [8] and common polymorphisms in the GR and other genes partially contribute to disparities in HPAA reactivity [17], [18], [19], [20].

There is increasing evidence for involvement of the epigenome in altering short and long-term status of GR and cortisol responsiveness [21]. The nerve growth factor-inducible protein A (NGFI-A), is a transcription factor that has been shown in the rat [22] and human [23] to regulate the expression of the NR3C1 promoter; its methylation down regulates gene expression [22]. In a seminal article, Weaver, Meaney and colleagues [21] showed that differential maternal care in rat pups modified the methylation pattern of the hippocampal GR exon 1_7_ which led to significant differences in subsequent adult behavior. Importantly, the cytosine residue within the 5′ CpG dinucleotide of the noncononocal NGFI-A (CpG_31_, CpG_32_) consensus sequence was highly methylated (associated with low GR expression) in the offspring of low caring mothers, and rarely methylated (high GR expression) in the offspring of high caring dams explaining the observed differences in HPAA reactivity in the adult offspring. The impact of maternal care on the epigenome is mediated by serotonergic (5-HT) neurotransmission that drives downstream expression of NGFI-A targeting its cognate binding site on the GR exon 1_7_ promoter. In humans, McGowan et al [23] showed that the hippocampal GR promoter 1F exon (orthologue to the rat exon 1_7_) was characterized by increased methylation of the NGFI-A transcription factor in suicide victims who were exposed to childhood abuse. Oberlander et al [24] showed that the cord blood neonatal GR methylation pattern including exon 1F was influenced by mother's mood and SSRI treatment during pregnancy. Increased GR methylation at this site was also associated with increased salivary cortisol stress responses at 3 months. Moser et al [25] found that in post-mortem hippocampal specimens from 32 subjects with various psychiatric disorders, a noncanonical site (equivalent to McGowan CpG_32_ as in the current study) was poorly methylated and the adjacent sites were unmethylated. They interpret their results as indicating that the mechanism presented by Weaver and associates is probably different between humans and rat. Similarly, Alt et al [26] showed low methylation levels in both major depressive disorder and control brains. Exon 1F was uniformly unmethylated. The variability in methylation effects across studies may reflect the fact that changes in methylation, which may be reversible [27], are also likely subject to multiple influences by both stressors and genetics. As noted by Meaney et al [28], the enzymatic machinery required to alter cytosine methylation is operative in post-mitotic neurons.

Prompted by these intriguing studies, and the importance of understanding the basis for individual and gender differences in HPAA reactivity with its profound implications for overall psychological and physical wellbeing, we used pyrosequencing [29] of bisulfite treated buccal DNA to assess the methylation status of the NR3C1 exon 1F promoter sequence in nonclinical subjects.

In addition, towards examining the role of genetic factors in understanding individual differences in HPAA response to social stress, we also genotyped these subjects for two salient genes, the serotonin transporter (*SLC6A4*) promoter region (5-HTTLPR) indel [30] and the estrogen receptor alpha (*ESR1*) promoter region TA repeat [31], respectively coding for serotonergic neurotransmission and a gonadal sex hormone that based on both animal and human studies, are known to modulate HPAA [8], [32], [33].

Our strategy in this investigation is to simultaneously model both epigenetic factors as well as sequence variations of two candidate genes towards understanding how nurture and nature modulate in a gender-specific manner HPAA response to social stress. Determination of both GR methylation in the exon 1F and genotyping polymorphic promoter-regions in two salient genes that impact the HPAA will allow integration and evaluation of the interactions between distinct trans polymorphisms, methylation and stress hormone response to social stressors. The strategy employed in the current investigation illustrates an important step towards deciphering the molecular determinants of complex phenotypes.

Estradiol especially seems to exert modulating effects on HPAA functioning, including HPAA responsiveness to glucocorticoid negative feedback [34]. The ESR1 is of particular interest since maternal behavior can be modulated by ESR1 expression and intriguingly, such effects are transmitted across generations. Specifically, Champagne et al [35], [36] demonstrated that high maternal care females exhibited significantly elevated levels of ESR1 mRNA compared with low maternal care females. Levels of estrogen receptor will determine the responsiveness to this hormone and ultimately regulate the efficiency of estrogen-mediated signaling and the biological and behavioral outcomes associated with estrogen. In rats, estradiol treatment significantly increased the evening elevation in cortisol and the stress-induced rise in cortisol [37]. The ability of estradiol to inhibit glucocorticoid negative feedback occurs specifically via the estrogen receptor α (ESR1), acting at the level of the paraventricular nucleus. Altogether estrogen plays an important role in HPAA regulation [38], [39]. Hence, we hypothesized that the polymorphic ESR1 gene is a worthwhile candidate, along with epigenetic modifications of the GR receptor, to impact social stress evoked increases in salivary cortisol especially in women subjects. ESR1 was of particular interest since this polymorphism seems to be a marker for important clinical outcomes in women [40].

There is considerable evidence for the idea that serotonin (5-HT) mediates the effect of postnatal modulation by differential maternal care on hippocampal GR expression since these effects are blocked by concurrent treatment with a serotonin receptor antagonist [41]. Importantly, 5-HT is not only a key regulatory neurotransmitter of HPAA responsiveness but also enables the methylation of the NGFI-A transcription factor, important in rat maternal care [42]. The serotonin transporter (SLC6A4) is an important element in regulation of synaptic serotonin levels [30] leading us to hypothesize in our model that the serotonin transporter, along with ESR1 (‘nature’), contribute jointly with epigenetic modification of GR (‘nurture), to explain individual differences in gender-sensitive stress responses, especially regarding gender differences.

The nonclinical subjects in the current study were tested for HPAA responsiveness to acute social stress [20] obtained by using a laboratory-based paradigm, the Trier Social Stress Test (TSST) [43], that leverages a ‘threat to the social self’ [2] via public speaking and mental arithmetic, to generate an unambiguous physiological endpoint, indexed by salivary cortisol. Importantly, both the TSST response and basal cortisol levels have been shown to be substantially heritable [44], [45], providing the necessary background for the current investigation.

## Results

### Methylation of GR exon 1F in men and women

**Figure 1:**
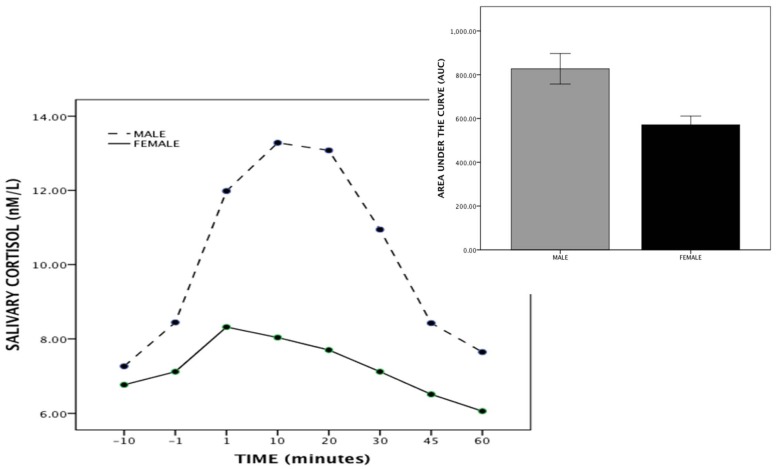
Salivary cortisol levels following the TSST. Area under the curve following the TSST for males and females. The rise in salivary cortisol is significant (SPSS GLM repeated measures) for men (F = 22.32 p<0.001) and for females (F = 9.04 p<0.001).

**Figure 2:**
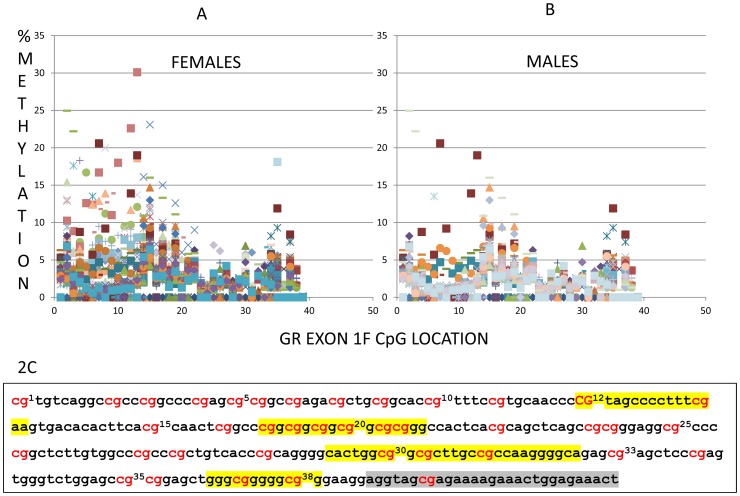
Methylation levels across 39 CpG sites in exon 1F for each subject. Methylation level of GR exon 1F at individual CpG sites for females (n = 46) (2A) and males (n = 46) (2B). Each point in the figure represents a single individual's percentage methylation value for each of the 39 CpG sites that were analyzed by bisulfite pyrosequencing. The points were color coded by the graph option in Excel and each color and shape represents an individual subject. The graph illustrates the marked individual differences (indicated by the shape and color of markers) in percentage methylation at each CpG site. Figure (2C) presents the promoter sequence of exon 1F showing the CpG sites and their position. Highlighted regions represent known or putative canonical (italics) and noncanonical (not italics) NGFI-A–binding sites, with the shaded grey area indicating the beginning of the exon (following McGowan et al (23)).

**Figure 3:**
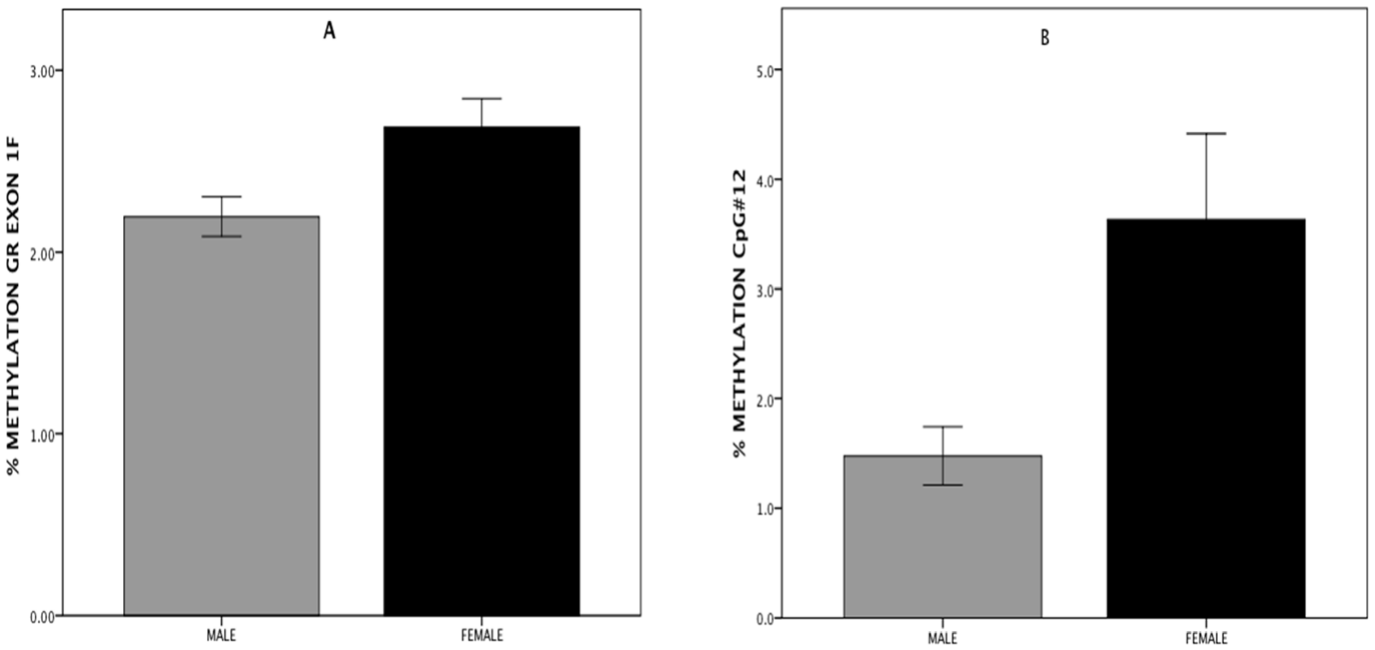
Methylation levels of the GR exon 1F and NFGI-A CpG #12 by gender. Percentage methylation levels of exon 1F (A) and NFGI-A CpG #12 (B) for males and females.

As previously reported by us [20] and others (9), in both men and women there is a significant increase (greater in males compared to females) in salivary cortisol levels following the TSST (Figure 1). The stress induced rise in salivary cortisol is presented in Figure 1 for each time point in a GLM repeated measures plot (SPSS) as well as AUC (see insert) for both men and women. There is a significant rise in cortisol (GLM repeated measures) for both men (tests of within subjects (F = 22.32, p<0.001; Huyhn-Feldt corrected) and women (F = 9.04, p<0.001; Huyhn-Feldt corrected). Since men and women are characterized by significantly different cortisol levels, each sex was analyzed separately and when sex is included in the analysis it is entered as an independent variable We next examined the methylation level and averaged the results across 39 assayed CpG sites in exon 1F for each subject (Figure 2). Overall, women showed significantly greater methylation levels than did men (Figure 3A) across the entire promoter region (t = 2.538, p = 0.013). Notably, marked individual differences for both men and women were observed at many individual CpG sites (Figure 2A, 2B). Overall levels of GR exon 1F methylation were similar to those previously observed by Oberlander et al [24] in peripheral tissue.

### GR exon 1F methylation and total cortisol output (AUC)

**Figure 4:**
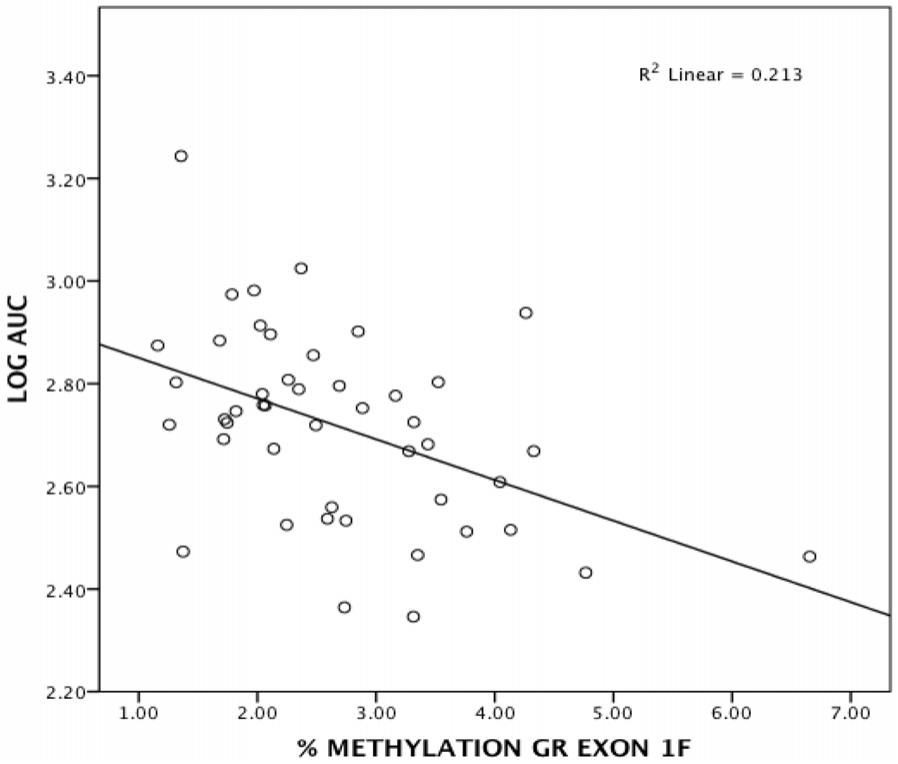
GR exon 1F methylation and total cortisol output. Correlation between AUC and average percent methylation at GR exon 1F in female subjects.

We next examined the relationship between sex, average methylation level across exon 1F, the interaction (sex x methylation) and AUC (summarized in Table 1). Sex (R^2^Δ = 0.116 F_1,89_ = 11.809, p = 0.001) and 1F methylation (R^2^Δ = 0.065 F_1,88_ = 7.082 p = 0.009) were significant predictors of AUC. The interaction between sex and exon 1F methylation did not attain significance (R^2^Δ = 0.197, F_1,88_ = 1.793, p = 0.184). Nevertheless, because of the potential role of sex differences as evidenced by sex differences in AUC, all subsequent analyses were carried out separately for men and women. For men, methylation was not a significant predictor (p = 0.722). In contrast, for women (Figure 4), the average methylation level of the GR 1F exon was inversely related to the amount of salivary cortisol secreted (AUC) during the TSST (R^2^Δ = 0.213 F_1,44_ = 11.877, p = 0.001), accounting for 21.3% of the variance.

**Table 1:** Linear regression analysis (average % methylation).

Dependent variable	AUC		
Regressor	(1)	(2) only Females	(3) only Females
Gender	−0.116** (0.042)		
Average % methylation 39 CpG sites	−0.059** (0.022)	−0.079** (0.023)	−.083*** (0.18)
Serotonin transporter (5-HTTLPR)			0.016** (0.005)
ESR1_dummy1 Estrogen alpha			−0.180** (0.063)
ESR1_dummy2 Estrogen alpha			−0.179** (0.054)
Intercept	3.107*** (0.075)	2.93*** (0.067)	2.956*** (0.075)
Adj. r square	0.163	0.196	0.506

* p<0.05; **p<0.01; ***p<0.001.

The significance levels are from the Coefficient's Table from the linear regression output of SPSS. The numbers in parentheses are the SE of the unstandardized beta coefficients. Each column is a separate regression analysis representing the addition of the predictor (column 1 = sex+methylation; column 2 = only in females methylation; column 3 is methylation + serotonin transporter + ESR1 dummy variables).

### Methylation of the noncanonical transcription factor NGFI-A CpG #12

Since this specific NGFI-A site displayed differential methylation, and was prognostic of behavior and clinical state both in the original rat study of maternal care [21] as well as the more recent human study of suicide with child abuse [23], we examined individual CpG sites within the NGFI-A recognition sequences as predictors of AUC (Table S1 for summary). A significant difference (Figure 3B) at CpG #12, a noncanonical NFGI-A binding site, was also observed in methylation between males and females (t = 2.618, p = 0.012). In the regression model both sex (R^2^Δ = 0.113, F_1,62_ = 7.923, p = 0.007) and CpG#12 (R^2^Δ = 0.173, F_1,61_ = 14.78, p<0.001) were significant predictors of AUC. There is no significant interaction between CpG#12 and sex (p = 0.652). Moreover, it is the only CpG site among the 39 tested that remains a significant predictor of AUC following Bonferroni correction (p_corrected_ = 0.03).

### ESR1 and 5-HTTLPR are independent predictors of AUC

All subjects were genotyped for two relevant genes, the ESR1 and 5-HTTLPR. We continued to analyze the proposed model (Table 1 for summary) in which average 1F methylation, 5-HTTLPR, ESR1 and the interaction between each gene and methylation level predict AUC (AUC = methylation + 5-HTTLPR + ESR1). In female subjects, there is a significant main effect of 5-HTTLPR (R^2^Δ = 0.172, F_1,42_ = 12.032, p = 0.001) and ESR1 (R^2^Δ = 0.132, F_2,40_ = 5.634, p = 0.007) on AUC. The interaction between 5-HTTLPR and 1F methylation did not attain significance (R^2^Δ<0.001, F_1,42_ = 0.019, p = 0.891). Nor was there any significant interaction between ESR1 x exon 1F methylation (p = 0.452). Altogether, in the full model (1F methylation, 5-HTTLPR and ESR1 polymorphisms) a total of 50.06% (adjusted R^2^) of the variance in total salivary cortisol output is explained. There was no significant effect of genotype on methylation.

## Discussion

A fuller understanding of the molecular mechanisms underlying differences between male and female response to stress has potentially profound implications for explaining gender differences in vulnerability to both psychopathology [46] and physical disease [47], [48], [49]. We have used a well-characterized laboratory based social stress test to examine the impact of epigenetic and genetic variation on cortisol response in a group of nonclinical subjects. In women, and not in men, the averaged methylation of 39 examined CpG sites located across the GR promoter exon 1F is a highly significant predictor of total cortisol response (AUC) in the TSST.

We specifically focused on a specific CpG (CpG#12) since this single CpG site is located in the exon 1F noncanonical NGFI-A transcription factor. Importantly, NGFI-A has been shown both in the rat and human to regulate the expression of the NR3C1 promoter; its methylation down regulates gene expression [21], [23] This specific CpG explains a notable 28% of the variance in total cortisol output in women. Importantly, women show significantly greater methylation in exon 1F, and at the NGFI-A transcription factor site, compared to men. That being said, our provisional results on a relatively small sample size should be considered exploratory and we eagerly await future replications in an independently recruited sample towards validating these first findings.

Gender effects in HPAA responsiveness are likely due to the cellular environment in women and men which differs substantially regarding the hormonal milieu and the impact of sex on the penetrance and expressivity of a wide variety of quantitative traits have been underscored [50]. Several studies have indicated that in the presence of estradiol there is an enhanced responsiveness of the HPAA to a stressor that may be due in part to impairment of the glucocorticoid negative feedback loop [37]. Additionally, in the rat model of high and low maternal care, there were differences in brain ESR1 mRNA levels between offspring of high and low mothers [35]. Hence, based on the information provided by the rat model of maternal care, we thought it worthwhile to examine the contribution of the ESR1 promoter-region repeat polymorphism as a predictor of total cortisol output. We observed a significant main effect between this gene and methylation of the GR 1F exon.

The serotonin transporter promoter-region indel was also examined as a predictor of cortisol output. Serotonin plays an important role in HPAA responsiveness [51], [52] and, by a pathway independent of the GR, this transmitter was shown to mediate the methylation of the NGFI-A transcription factor in the rat model of high and low maternal care [42]. We confirm both a significant main effect of 5-HTTLPR short allele on basal cortisol [53], as well as a significantly increased overall cortisol rise (AUC) in carriers of the short allele following the TSST [54]. Similarly, in girls aged 9–14 exposure to a laboratory stress test, evoked greater cortisol response in subjects genotyped for the 5-HTTLPR short alleles [55]. Future investigations could profitably examine methylation patterns on other genes in the human stress response. Of especial interest is the serotonin transporter, since studies have observed salient epigenetic signatures in the serotonin transporter [56], [57], [58].

We observed that GR methylation and ESR1 and 5-HTTLPR genotypes independently predict AUC. The absence of a significant interaction between 5-HTTLPR and the ESR1 and 1F methylation in the regression analysis suggests that these two genes, which are known to partially mediate stress, are affecting the stress response independently of methylation of the GR. Our results might suggest that other pathways, and not solely GR exon 1F methylation, are mediating the effect of these hormones on HPAA responsiveness. For example, chronic estrogen administration has been shown to increase corticotrophin releasing hormone (CRH) mRNA levels, CRH synthesis and release in CRH-containing neurons of the paraventricular nucleus [59]. Indeed the human CRH gene contains functional half palindromic estradiol responsive element [60] and estrogen receptors are considered important modulators of CRH secretion [61]. Another mechanism for ESR1 impacting HPAA responsiveness without directly impacting the GR receptor methylation profile, is based on the report of Westberg et al [31] that ‘neuroticism’, ‘psychoticism’, and ‘non-conformity’ all appeared to be associated with the ER alpha gene. Hence, the effect of ER alpha on cortisol output might be mediated indirectly by personality traits.

Yet an additional explanation is suggested by the work of Turner's group [16]. They showed in white blood cells of healthy donors, highly variable methylation patterns among different GR promoters, suggesting that epigenetic programming may not be restricted to the 1F promoter, but operates throughout the extended CpG island [16]. It cannot, therefore, be excluded that the impact of estrogen and serotonin is mediated by other GR first exons and not solely by 1F. In such a scenario no statistic interaction would be expected between GR 1F methylation and either of the two polymorphic genes we examined. Clearly, future studies should examine the methylation patterns of other first exon alternative splicing forms with the aim to clarify their relationship to HPAA responsiveness.

An increasing volume of experimental research also indicates that 5HT can modulate HPAA responsiveness and cortisol release beyond direct actions at the level of the GR [51]. For example, Heisler et al have shown that 5HT_2C_ receptors in the paraventricular nucleus of the hypothalamus mediate serotonin-induced release of CRH. Additionally, serotonin directly induces secretion of cortisol directly from the human adrenal gland [62]. Finally, Moser et al [63] have recently shown by PET imaging that release of cortisol by serotonin is likely mediated by 5HT_1A_ receptor located in the hypothalamus and are known to mediate the release CRH [64]. These findings may help to understand the current finding that the serotonin transporter polymorphism has a main effect on HPAA responsiveness but shows no significant statistical interaction with the GR exon 1F methylation [61].

Failure to observe an interaction could also be an issue of power. Although the sample size in the current study is relatively large in comparison to the few studies of GR methylation and behavior so far carried out in humans, further stratifying the female subjects by ESR1 repeat length ipso facto reduces power. So we cannot rule out the possibility that failure to observe a significant interaction in the regression analysis is simply a power issue. The current results cannot distinguish between these various alternative explanations leaving such explanations to be resolved in future investigations.

Interestingly, in the rat model it has been shown that NGFI-A is required for serotonin-induced DNA demethylation and increased exon 1_7_ expression [22]. Intriguingly, the 5-HTTLPR polymorphism is controversially linked to depression mediated by stressful life events [65], [66], [67], [68] and it has recently been suggested that the method used to assess environmental adversity, as well as clinical assessment of the subjects, could explain most discrepancies in results [65]. Studies using objective evidence or detailed interviews to assess environmental adversity in context consistently find an interaction in the expected direction, but studies relying on brief self-report measures of adversity often showed negative findings. Our findings suggest the notion that a possibly objective measure of stressful life events that could be incorporated in G×E studies, such as pioneered by Caspi and his colleagues [68], [69], would be to examine methylation patterns of the GR receptor as we and others [23], [24], [25], [26] have done. After all, stressful life events need to impact the genome at the molecular level towards effecting behavior mediated presumably by modifying gene expression.

Ideally, research on methylation with regards to stress reaction would have been performed on brain tissue especially the hippocampus [21], [23]. Such studies in people, however, are clearly restricted to the availability of post-mortem material and constrained by particular clinical diagnoses [70]. In the current study we instead examined a peripheral tissue, buccal epithelial cells, to monitor GR methylation status allowing us access to a much wider range of subjects. Some information regarding the relationship between buccal cells, white blood and brain cells is provided by Kaminsky et al [71]. Caution is needed in interpreting peripheral cells as models of brain function and it is not completely clear to what extent results in buccal cells can be extrapolated to brain despite their shared ectodermal origin. Peripheral tissues have been suggested as models or probes for studying human behavior [72] and there is evidence that gene expression in lymphocytes, for example, may partially reflect similar brain activity [73]. To our knowledge such analyses have not been carried out between buccal cells and brain.

Nevertheless, regardless of the relationship between buccal, brain and white blood cells, the peripheral GR has also been suggested to be important in its own right in the maintenance of HPAA homeostasis. In fact, there are well-established peripheral pathways that bypass or complement the HPAA [74]. For example, an increasing number of preclinical and clinical studies report dissociation of ACTH and cortisol levels in critical illness, inflammation and mental disorders [75]. Mechanisms involve altered adrenal responsiveness, aberrant receptor expression or modulation of adrenal function by cytokines, vasoactive factors or neuropeptides [75]. The current results highlight the importance of peripheral GR methylation as an important component in regulation of cortisol secretion.

It is tempting to hypothesize that in the presence of low GR concentration (high exon1F methylation [23]) only low levels of cortisol are required to activate peripheral tissues. HPAA responsiveness in such individuals is fine-tuned to the levels not only of hippocampal [23] and pituitary [76] glucocorticoid receptor expression but also, as suggested by the current findings, to the peripheral GR. Expression of the GR in peripheral tissues may in part be determined by the methylation status of the 1F exon. This notion is supported by our finding that when basal cortisol is entered as the dependent variable in the regression analysis similar results (GR methylation is a significant predictor of basal cortisol levels in women) are obtained as observed for AUC following the TSST. This suggests that increased cortisol levels are associated with decreased GR expression driven perhaps by increased GR promoter methylation. Such an explanation is consistent with GR downregulating its own receptor [77]. Of course, a limitation of the current study is that we did not measure levels of GR mRNA in the buccal washes and therefore we can only speculate regarding the role of methylation status on expression of this receptor.

A cardinal question raised by the current findings is the developmental window when the methylation pattern of GR buccal DNA is inscribed. Is the methylation pattern fixed in the first few weeks or months as observed by Oberlander et al [24] or perhaps represents years of environmental input? The current investigation is a cross-sectional study and cannot answer this important question and we can therefore only speculate regarding this issue. Clearly, longitudinal studies from early developmental times are required to resolve this questioin. In either scenario, the endpoint of these dynamic environmental challenges coupled with DNA sequence variations represented by common polymorphisms appear to explain a good percentage of individual and gender differences in HPAA responsiveness to social stress. By modeling in the current investigation epigenetic and genetic information we incrementally add to understanding how nature and nature jointly contribute to gender-specific responses to social stress.

## Methods

### For detailed methods see Methods S1

As described in a previous paper [20], participants were primarily college students from the Hebrew University. Selection criteria stipulated that subjects were <35 years old, had no history of psychiatric or endocrine illness, were currently non-smokers, were not pregnant, had not given birth in the past year, and were not using medication on a regular basis besides single-phase oral contraceptives. Altogether 92 subjects (46 males and 46 females, average age 25.29, S.D. = 3.6) were included in the study. The study was approved by the IRB of Herzog Hospital, Jerusalem and all subjects provided written informed consent. The subjects were tested for HPAA responsiveness to acute social stress [20] the Trier Social Stress Test (TSST) [43], that leverages a ‘threat to the social self’ [2] via public speaking and mental arithmetic, to generate an unambiguous physiological endpoint, indexed by salivary cortisol. Salivary cortisol was samples eight times. DNA was extracted from 20 ml of mouthwash samples using the Master Pure kit (Epicentre,Madison,WI). Pyrosequencing [29] of bisulfite treated buccal DNA was carried out by EpigenDX. In addition, we genotyped these subjects for two salient genes, the serotonin transporter (*SLC6A4*) promoter region (5-HTTLPR) [30] and the *ESR1* promoter region TA repeat [31]. Altogether, we examined the methylation level across 39 CpG sites in GR exon 1F promoter sequence for each subject. All statistical tests were carried out using SPSS version 15. A linear regression model was used to ascertain the effects of sex, GR methylation level and genes on stress response.

## Supporting Information

Table S1 **Linear regression analysis (noncanonical NGFI-A CpG#12).**
(DOC)

Methods S1 (DOC)
