# Supplementary material for: Epigenetic and Genetic Factors Predict Women's Salivary Cortisol following a Threat to the Social Self
Source: PLoS One. 2012 Nov 14;7(11):e48597. doi: 10.1371/journal.pone.0048597 (PMC3498240; doi:10.1371/journal.pone.0048597)
Supplement: Table S1 — Linear regression analysis (noncanonical NGFI-A CpG#12). (DOC) [file pone.0048597.s001.doc]

Supplementary Table 1. Linear regression analysis (noncanonical NGFI-A CpG#12)

| Dependent variable | AUC | | |
| --- | --- | --- | --- |
| Regressor | (1) | (2)  only Females | (3)  only Females |
| Gender | -0.107*  (0.049) |  |  |
| % methylation CpG #12  NGFI-A | -0.022**  (0.006) | -0.022 **  (0.006) | -0.325**  (0.054) |
| Serotonin transporter (5-HTTLPR) |  |  | 0.015*  (0.006) |
| ESR1_dummy1  Estrogen alpha |  |  | -0.131  (0.079) |
| ESR1_dummy2  Estrogen alpha |  |  | -0.172**  (0.060) |
| Intercept | 3.018***  (0.077) | 2.806***  (0.036) | 2.614***  (0.125) |
| Adj. r square | 0.248 | 0.260 | 0.498 |
| *p<0.05; **p<0.01; ***p<0.001 | | | |

The significance levels are from the Coefficient’s Table from the linear regression output of SPSS. The numbers in parentheses are the SE of the unstandardized beta coefficients. Each column is a separate regression analysis representing the addition of the predictor (column 1=sex+methylation; column 2 = only in females methylation; column 3 is methylation + serotonin transporter + ESR1 dummy variables).
