# Supplementary material for: Epigenetic and Genetic Factors Predict Women's Salivary Cortisol following a Threat to the Social Self
Source: PLoS One. 2012 Nov 14;7(11):e48597. doi: 10.1371/journal.pone.0048597 (PMC3498240; doi:10.1371/journal.pone.0048597)
Supplement: Methods S1 — (DOC) [file pone.0048597.s002.doc]

**Supplementary Information**

**Methods**

**1.1 Subjects and TSST**

[1] Participants were primarily college students at Israeli institutions of tertiary education recruited by word of mouth and advertisements on campus notice boards for a study on human genetics and personality. Selection criteria stipulated that subjects were <35 years old, had no history of psychiatric or endocrine illness (conﬁrmed by a screening interview but not a structured interview), were currently non-smokers, were not pregnant, had not given birth in the past year, and were not using medication on a regular basis besides single-phase oral contraceptives. Altogether 92 subjects (46 males and 46 females, average age 25.29, S.D. = 3.6) were included in the study.

The study was approved by the IRB of Herzog Hospital, Jerusalem and all subjects provided written informed consent. Subjects received a modest monetary incentive for participation.

General procedure: Prior to the testing session, subjects were given two sterile test tubes, each containing 10 ml of Aquafresh mouthwash, for DNA sampling. DNA mouthwashes were returned by mail or hand-delivered to our lab. After receipt of their DNA, subjects were scheduled for testing within a ﬁxed time-window (between 1500 and 1800 h) to counter effects of circadian changes in cortisol. To limit variance, subjects were given explicit instructions to refrain from excessive physical activity for 2 h prior to the experiment and from brushing their teeth, eating, and drinking (besides water) for the 90 min prior to the testing session. Testing was carried out in a laboratory in the Department of Psychology, Hebrew University. Prior to testing, subjects were asked about their present health and whether they had taken medication (besides oral contraception) during the previous 24 h. Those who described themselves as ill or medicated were rescheduled.

The TSST paradigm entails two parts (5 min each), free speech and a mental arithmetic task performed in front of two stone-faced interviewers and a camera [2]. Salivary cortisol was sampled eight times during the 90 min. TSST session at the following time-points: 10 min. prior to testing, 1 min. prior to testing, immediately after testing, and 10, 20, 30, 45, and 60 min. post-test.

As discussed in a previous study by our group [1] stage of the menstrual cycle has been reported to impact salivary cortisol rises in the TSST paradigm [2]. Hence, we compared cortisol measures obtained from women who were using oral contraception (OC+) versus women who were not using oral contraception (OC-), and then compared the cortisol measures of each group of women to men. None of the analyses yielded signiﬁcant results. Additionally, both groups of women differed signiﬁcantly from the men on the same measures, and the values obtained from men were higher than those of the women. Based on these results, contraceptive-use was not considered in subsequent analyses.

**1.2. DNA extraction and genotyping**

DNA was extracted from 20 ml of mouthwash samples using the Master Pure kit (Epicentre,Madison,WI).

Amplification of the ESR1 microsatellite was achieved using the following primers (corresponds to the TA dinucleotide repeat in the 5’ promoter region): forward (fluorescent) 5’- AGACGCATGATATACTTCACC–3’;reverse5’- GTTCACTTGGGCTAGGATAT -3’. PCR reactions were performed using 5µl Master Mix (Thermo scientific), 0.5µl primers (0.5 µM), 0.4µl Mg/Cl2 (2.5 mM) and 3.1µl of water to total of 9µl total volume and an additional 1µl of genomic DNA was added to the mixture. All PCR reactions were employed on a Biometra T1 Thermocycler (Biometra, Güttingem, Germany). PCR reaction conditions were as follows: preheating step at 95.0˚C for 5 min, 30 cycles of denaturation at 95.0˚C for 30 s, reannealing at 55˚C for 30 s and extension at 72˚C for 40 s. The reaction proceeded to a hold at 72˚C for 10 min. The PCR product was analyzed on an ABI 310 DNA Analyzer. The 5-HTTLPR polymorphism (44bp deletion/insertion) in the promoter region was characterized by a PCR amplification procedure with the following primers: F5'-GGCGTTGCCGCTCTGAATTGC-3';

R5'-GAGGGACTGAGCTGGACAACC-3'. PCR reactions were performed using 5µl Master Mix (Thermo scientific), 2µl primers (0.5 µM), 0.6µl Mg/Cl2 (2.5 mM), 0.4µl DMSO 5% and 1µl of water to total of 9µl total volume and an additional 1µl of genomic DNA was added to the mixture. All PCR reactions were employed on a Biometra T1 Thermocycler (Biometra, Güttingem, Germany). PCR reaction conditions were as follows: Preheating step at 94.0˚C for 5 min, 34 cycles of denaturation at 94.0˚C for 30 s, reannealing at 55˚C for 30 s and extension at 72˚C for 90 s. The reaction proceeded to a hold at 72˚C for 5 min. All reaction mixtures were electrophoresed on a 3% agarose gel (AMRESCO) with ethidium bromide to screen for genotype.

**1.3. Bisulfite treatment and methylation analysis:**

Pyrosequencing of bisulfite-treated DNA was carried out by EpigenDx (<http://www.epigendx.com/Methylation.html>). Pyrosequencing for allele quantification (PSQ H96A, Qiagen Pyrosequencing) is a real-time sequencing-based DNA analysis that quantify multiple, and consecutive CpG sites individually as artificial T/C SNPs.

Bisulfite modification was carried out using Zymo Research EZ Methylation kit (Zymo research, Orange, CA). Briefly, 500 ng of sample DNA was bisulfate treated followed by PCR amplification in accordance with the manufacturer’s instructions (<http://www.zymoresearch.com/content/ez-96-dna-methylation-kit-d5003>). Succinctly, the DNA was denatured with a 5μl dilution buffer incubated overnight at 50 °C with CT conversion reagent, followed by a clean-up, desulfonation, and elution. The bisulfite-modified DNA was used immediately for PCR or stored at –70 °C.

Human GC methylation assays cover thirty-nine CG dinucleotides in the promoter region ranging from -630 ~ -354 from the transcriptional start site based on Ensemble ID ENST00000231509. The sequence through every CpG site in a 300 bp region, three PCR assays and five pyrosequencing assays were designed and tested for PCR preferential amplification (EpigenDx Inc., Worcester, MA). The PCR was performed using 10X PCR buffer, 3.0 mM MgCl_2_ , 200 μM of each dNTP, 0.2 µM each of forward and reverse primers, HotStar DNA polymerase (Qiagen Inc.) 1.25 U, and ~10 ng of bisulfite converted DNA per 30 μl reaction. PCR cycling conditions were: 95 °C for 15 min, 45 cycles of 95 °C for 30s, 46 ºC or 56ºC for 30 s, and 72 °C for 30 s, followed by an extension at 72 °C for 5 min and then products were held at 4 °C.

The PCR was performed with one of the PCR primers biotinylated to convert the PCR product to single-stranded DNA templates. The PCR products were sequenced by pyrosequencing PSQ96 HS System (PSQ H96A, Qiagen Pyrosequencing) following the manufacturer’s instructions (PSQ H96A, Qiagen Pyrosequencing). The methylation status of each locus was analyzed individually as a T/C SNP using QCpG software (PSQ H96A, Qiagen Pyrosequencing).

In order to validate the accuracy of the quantitative pyrosequencing results, a preferential amplification testing of for each PCR assay was tested using unmethylated DNA control and in vitro methylated DNA, which were mixed at different ratios followed by bisulfite modification, PCR and pyrosequencing analysis. The methylation level obtained from the mixing study was highly correlated with expected methylation percentages with an R² > 0.9.

**1.4. Statistical analysis**

All statistical tests were carried out using SPSS (PASW STATISTICS 18.0, Windows). We calculated area under the curve (AUC) for cortisol. We used the formula for AUC with respect to ground (AUCg) [3], based on the trapezoid formula, to reflect total output for cortisol. This formula is given by


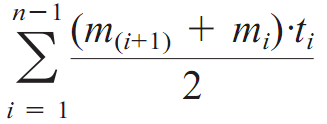


where n = the number of samples, t_i_ is the interval between sample i and sample i+1 and m_i_  is the level of the biomarker for sample i. This score incorporates information regarding both baseline and responsiveness within one score and thus allows us to maximize information being entered into regression equations while sacrificing few degrees of freedom. As noted by Preussner and his colleagues [3], with endocrinological data, it can be assumed that the use of the AUCg will result in a measure that is more related to ‘total hormonal output’ (see [4,5,6]).

The relationship between methylation and AUC following the TSST was examined by SPSS linear regression modeling. Statistical normality of the data was checked using the Kolmogorov–Smirnov test. The distributions of AUC were skewed; they were log10 transformed and became normally distributed. The AUC was the dependent variable and sex, methylation level (percentage of methylated regions) of the GR exon 1F region, and the two genetic polymorphisms (ESR1 and 5-HTTLPR) were the predictors. We evaluated the proportion of variance in the dependent measure (AUC) accounted for by predictors (sex and methylation level) and the significance of the increment in variance (R^2^Δ) accounted for by each predictor. Because of the role of sex differences in stress responses, men and women were examined separately.

References:

1. Shalev I, Lerer E, Israel S, Uzefovsky F, Gritsenko I, et al. (2009) BDNF Val66Met polymorphism is associated with HPA axis reactivity to psychological stress characterized by genotype and gender interactions. Psychoneuroendocrinology 34: 382-388.

2. Kirschbaum C, Kudielka BM, Gaab J, Schommer NC, Hellhammer DH (1999) Impact of gender, menstrual cycle phase, and oral contraceptives on the activity of the hypothalamus-pituitary-adrenal axis. Psychosom Med 61: 154-162.

3. Pruessner JC, Kirschbaum C, Meinlschmid G, Hellhammer DH (2003) Two formulas for computation of the area under the curve represent measures of total hormone concentration versus time-dependent change. Psychoneuroendocrinology 28: 916-931.

4. Chopra KK, Ravindran A, Kennedy SH, Mackenzie B, Matthews S, et al. (2009) Sex differences in hormonal responses to a social stressor in chronic major depression. Psychoneuroendocrinology.

5. Wessa M, Rohleder N, Kirschbaum C, Flor H (2006) Altered cortisol awakening response in posttraumatic stress disorder. Psychoneuroendocrinology 31: 209-215.

6. Lederbogen F, Kuhner C, Kirschbaum C, Meisinger C, Lammich J, et al. (2010) Salivary cortisol in a middle-aged community sample: results from 990 men and women of the KORA-F3 Augsburg study. Eur J Endocrinol 163: 443-451.
